# Exercise training and α_1_‐adrenoreceptor‐mediated sympathetic vasoconstriction in resting and contracting skeletal muscle

**DOI:** 10.14814/phy2.12707

**Published:** 2016-02-11

**Authors:** Timothy P. Just, Darren S. DeLorey

**Affiliations:** ^1^ Faculty of Physical Education and RecreationUniversity of AlbertaEdmontonABCanada

**Keywords:** Sympathetic nervous system, vasoconstriction, exercise training

## Abstract

Exercise training (ET) increases sympathetic vasoconstrictor responsiveness and enhances contraction‐mediated inhibition of sympathetic vasoconstriction (i.e., sympatholysis) through a nitric oxide (NO)‐dependent mechanism. Changes in α_2_‐adrenoreceptor vasoconstriction mediate a portion of these training adaptations, however the contribution of other postsynaptic receptors remains to be determined. Therefore, the purpose of this study was to investigate the effect of ET on α_1_‐adrenoreceptor‐mediated vasoconstriction in resting and contracting muscle. It was hypothesized that α_1_‐adrenoreceptor‐mediated sympatholysis would be enhanced following ET. Male Sprague Dawley rats were randomized to sedentary (S; *n* = 12) or heavy‐intensity treadmill ET (*n* = 11) groups. Subsequently, rats were anesthetized and instrumented for lumbar sympathetic chain stimulation and measurement of femoral vascular conductance (FVC) at rest and during muscle contraction. The percentage change in FVC in response to sympathetic stimulation was measured in control, α_1_‐adrenoreceptor blockade (Prazosin; 20 μg, IV), and combined α_1_ and NO synthase (NOS) blockade (l‐NAME; 5 mg·kg^−1^ IV) conditions. Sympathetic vasoconstrictor responsiveness was increased (*P *< 0.05) in ET compared to S rats at low, but not high (*P *> 0.05) stimulation frequencies at rest (S: 2 Hz: −25 ± 4%; 5 Hz: −45 ± 5 %; ET: 2 Hz: −35 ± 7%, 5 Hz: −52 ± 7%), whereas sympathetic vasoconstrictor responsiveness was not different (*P *> 0.05) between groups during contraction (S: 2 Hz: −11 ± 8%; 5 Hz: −26 ± 11%; ET: 2 Hz: −10 ± 7%, 5 Hz: −27 ± 12%). Prazosin blunted (*P *< 0.05) vasoconstrictor responsiveness in S and ET rats at rest and during contraction, and abolished group differences in vasoconstrictor responsiveness. Subsequent NOS blockade increased vasoconstrictor responses (*P *< 0.05) in S at rest and during contraction, whereas in ET vasoconstriction was increased (*P *< 0.05) in response to sympathetic stimulation at 2 Hz at rest and unchanged (*P *> 0.05) during contraction. ET enhanced (*P *< 0.05) sympatholysis, however the training‐mediated improvements in sympatholysis were abolished by α_1_‐adrenoreceptor blockade. Subsequent NOS inhibition did not alter (*P *> 0.05) sympatholysis in S or ET rats. In conclusion, ET augmented α_1_‐adrenoreceptor‐mediated vasoconstriction in resting skeletal muscle and enhanced α_1_‐adrenoreceptor‐mediated sympatholysis. Furthermore, these data suggest that NO is not required to inhibit α_2_‐adrenoreceptor‐ and nonadrenoreceptor‐mediated vasoconstriction during exercise.

## Introduction

Our laboratory recently reported that exercise training alters sympathetic vasoconstrictor responsiveness (the magnitude of the decrease in femoral vascular conductance in response to stimulation of the lumber sympathetic chain) (Jendzjowsky and DeLorey 2012) and enhances skeletal muscle contraction‐mediated inhibition of sympathetic vasoconstriction (sympatholysis) (Jendzjowsky and DeLorey 2013b). Pharmacological inhibition of nitric oxide synthase (NOS) demonstrated that the training‐induced improvement in sympatholysis was mediated by nitric oxide (NO) (Jendzjowsky and DeLorey 2013b). NO may open ATP‐sensitive K^+^ channels and hyperpolarize vascular smooth muscle leading to a closure of voltage‐gated Ca^2+^ channels and inhibition of α_2_‐adrenoreceptor‐mediated vasoconstriction that is dependent on an influx of extracellular Ca^2+^ through voltage‐gated Ca^2+^ channels (Tateishi and Faber 1995; Thomas et al. 1997).

However, we recently tested the hypothesis that enhanced NO‐mediated inhibition of sympathetic vasoconstriction following exercise training was α_2_‐adrenoreceptor dependent. Our study demonstrated that selective α_2_‐adrenoreceptor blockade did not alter sympatholysis in sedentary and mild‐intensity exercise‐trained rats and only modestly reduced sympatholysis in heavy‐intensity exercise‐trained rats. These data indicate that blunting of α_2_‐adrenoreceptor‐mediated vasoconstriction made only a small contribution to the enhanced sympatholysis following heavy‐intensity exercise training (Jendzjowsky and DeLorey 2013a). In the presence of α_2_‐adrenoreceptor blockade, NOS inhibition reduced the magnitude of contraction‐mediated inhibition of sympathetic vasoconstriction during high‐frequency sympathetic nerve stimulation in heavy‐intensity exercise‐trained rats, suggesting that exercise training enhanced NO‐mediated inhibition of α_1_‐adrenoreceptor‐ and/or nonadrenoreceptor‐mediated sympathetic vasoconstriction (Jendzjowsky and DeLorey 2013a).

Previous studies (Buckwalter et al. 2001; Rosenmeier et al. 2003; Wray et al. 2004) have demonstrated that both α_1_‐ and α_2_‐adrenoreceptors contribute to sympatholysis and NO‐mediated inhibition of α_1_‐adrenoreceptor‐mediated vasoconstriction has been documented (Ohyanagi et al. 1992; Tuttle and Falcone 2001; Ives et al. 2012). However, whether aerobic exercise training alters α_1_‐adrenoreceptor‐mediated vasoconstriction in resting and contracting muscle has not been investigated.

Therefore, the purpose of this study was to investigate the effect of selective α_1_‐adrenoreceptor blockade on sympathetic vasoconstrictor responsiveness and NO‐mediated sympatholysis. It was hypothesized that selective α_1_‐adrenoreceptor blockade would abolish the enhanced sympatholysis in exercise‐trained rats demonstrating that sympatholysis is enhanced following exercise training by greater blunting of α_1_‐adrenoreceptor‐mediated vasoconstriction. It was also hypothesized that in the presence of α_1_‐adrenoreceptor blockade, NOS inhibition would not alter sympatholysis demonstrating that NO inhibits vasoconstriction through a α_1_‐adrenoreceptor‐dependent mechanism and that NO is not required to inhibit α_2_‐adrenoreceptor‐ and nonadrenoreceptor‐mediated vasoconstriction in contracting muscle.

## Methods

### Animals and animal care

Male Sprague Dawley rats were obtained from the institutional breeding colony and housed in pairs in a 12:12‐h light–dark cycle, and environmentally controlled (22–24°C, 40–70% humidity) room. Water and rat chow (Lab Diet 5001, PMI Nutrition, Brentwood, MO) were freely available. All experiments were conducted in accordance with the Canadian Council on Animal Care Guidelines and Policies with approval from the Animal Care and Use Committee: Health Sciences for the University of Alberta.

### Chronic endurance exercise training

All rats were habituated to the laboratory and exercise by walking on a motorized treadmill (Panlab LE8710, Barcelona, Spain) 10 min day^−1^ for 5 days at 10 m min^−1^, 0° grade. Following familiarization, rats were randomly assigned to a sedentary time–control (*n* = 12) or exercise‐trained (*n* = 11) group. Exercise‐trained rats ran on a treadmill 5 days  week^−1^ for 4 weeks for a distance of 600 m at 40 m min^−1^and 5° grade each training session. Sedentary rats were handled and weighed daily. On the first day of training, rats in the exercise‐trained group completed 15, 1 min intervals at 40 m min^−1^ 5° grade interspersed with 1 min rest periods. Each subsequent training day, run time was increased while rest time was maintained. Within 2 weeks of the initiation of training, all exercise‐trained rats were able to run continuously for 600 m at the prescribed speed and grade. This training paradigm has been shown to increase heart mass, heart‐to‐body mass ratio, soleus citrate synthase activity, and endothelium‐dependent vasodilation (Jendzjowsky and DeLorey 2011, 2012, 2013b).

### Instrumentation

Approximately 24 h after the last training session anesthesia was induced by inhalation of isoflurane (3–3.5%, balance O_2_). The right jugular vein was cannulated and anesthesia was maintained by infusion of α‐chloralose (8‐16 mg kg^−1^ h^−1^) and urethane (50‐100 mg kg^−1^ h^−1^). The depth of anesthesia was assessed by the stability of arterial blood pressure, heart rate (HR), and the absence of a withdrawal reflex in response to painful stimuli (i.e., paw‐pinch). Core temperature was monitored by rectal probe and maintained at 36–37°C by an external heating pad (Physitemp, TCAT‐2, Clifton, NJ). A tracheotomy was performed to allow spontaneous breathing of room air. We have previously demonstrated the maintenance of arterial blood gases and acid base status at rest and during contraction in this preparation (Jendzjowsky and DeLorey 2013b); thus, arterial blood gases and acid base status were checked periodically to confirm the maintenance of normal values in these experiments (PaO_2_: 88–95 mmHg; PaCO_2_: 39–41 mmHg pH: 7.39–7.42). The left brachial artery was cannulated and connected to a solid state pressure transducer (Abbott, North Chicago, IL) for the continuous measurement of arterial blood pressure. Mean arterial pressure (MAP) and HR were derived from the arterial blood pressure waveform. The left femoral artery and vein were cannulated for the delivery of pharmacology. Blood flow was measured using a transit‐time flow probe (0.7V; Transonic Systems, Ithaca, NY) placed around the right femoral artery and connected to a flow meter (T106 Transonic Systems, Ithaca, NY).

### Muscle contraction

The right sciatic nerve was exposed and instrumented with a cuff electrode. The triceps surae muscle group was then dissected free of all skin and connective tissue and attached to a force transducer (Model FT03, Grass Technologies, Warwick, RI) via the calcaneal tendon. Hindlimb contractions were produced by electrical stimulation of the sciatic nerve with Chart 7.2^™^ software (AD Instruments, Colorado Springs, CO). The motor threshold (MT) and the optimal muscle length for tension development were determined. Maximal contractile force (MCF) was determined by stimulation of the triceps surae muscle group with 25, 1 msec impulses delivered at 100 Hz, 10× MT (motor threshold). The triceps surae muscles were stimulated (40 Hz, 0.1 msec pulses in 250 msec trains, at a rate of 60 trains per min at ~6× MT) to contract rhythmically at 60% MCF.

### Lumbar sympathetic chain stimulation

A laparotomy was performed and the aorta and vena cava were temporarily retracted to attach a bipolar silver‐wire‐stimulating electrode to the lumbar sympathetic chain at the L3/L4 level. The electrode was embedded and electrically isolated in a rapidly curing nontoxic silicone elastomer (Kwiksil, WPI, Sarasota, FL). The electrode was used to deliver constant current stimulations through an isolated stimulator (Digitimer DS3, Welwyn City, UK). Following a 20‐min stabilization period the following experiments were conducted.

### α_1_‐Adrenoreceptor vasoconstriction in resting and contracting skeletal muscle

The vasoconstrictor response to 1 min of lumbar sympathetic chain stimulation (1 msec, 1 mAmp pulses) delivered at 2 and 5 Hz in random order was measured at rest and during muscle contraction under control conditions, following the injection of the selective α_1_‐adrenoreceptor antagonist Prazosin (20 *µ*g bolus, IV) and during subsequent NOS blockade (combined Prazosin + l‐NAME, 5 mg kg^−1^, IV). Under resting conditions, efferent sympathetic nerve activity discharge frequencies are relatively low (∼1 Hz), with increases in firing frequency and neurotransmitter release occurring in response to exercise (Macefield et al. 1994; Johnson and Gilbey 1996; Hudson et al. 2011). In the present study, the lumbar sympathetic chain was stimulated at frequencies of 2 and 5 Hz to produce two distinct levels of sympathetic nerve activity reflective of sympathetic nerve activity at rest and during exercise and to evoke frequency‐dependent vasoconstrictor responses in the hindlimb vascular bed at rest and during muscle contraction.

Rhythmic muscle contraction was produced for 8 min and lumbar sympathetic chain stimulation was delivered 3 and 6 min after the onset of contraction in random order. Control, Prazosin, and combined Prazosin + l‐NAME conditions were separated by 30 min of recovery.

Upon completion of all experiments, animals were euthanized by an overdose of α‐chloralose and urethane and the heart was dissected free for measurement of cardiac mass.

### Effectiveness of α_1_‐adrenoreceptor blockade

The effectiveness and selectivity of α_1_‐adrenoreceptor blockade was assessed by injection of the selective α_1_‐adrenoreceptor phenylephrine (0.1 μg mL^−1^ min^−1^, IA) and the selective α_2_‐adrenoreceptor agonist, clonidine (0.1 μg mL^−1^ min^−1^, IA) prior to and following Prazosin.

### Pharmacology

All drugs were purchased from Sigma‐Aldrich (Oakville, ON, Canada) and dissolved in 0.9% physiological saline.

### Data analysis

Data were recorded using Chart software (AD Instruments, Colorado Springs, CO, USA). Arterial blood pressure and femoral artery blood flow (FBF) were sampled at 100 Hz and femoral vascular conductance (FVC) was calculated as FBF ÷ MAP (mL min^−1^ mmHg^−1^). Muscle force production was measured continuously and peak force development was determined for each muscle contraction. To compare force production between groups and experimental conditions, mean peak contractile force was calculated from minutes 3 to 7 (the time period encompassing the sympathetic stimulations) of each contractile bout.

The change in HR, MAP, FBF, and FVC in response to sympathetic stimulation was calculated as an absolute change and as a percentage change from the value preceding the sympathetic stimulation in Control, Prazosin, and combined Prazosin + l‐NAME conditions. The percentage change in FVC is the accepted metric to assess the magnitude of sympathetic vasoconstrictor responses because the percentage change in FVC accurately reflects percentage changes in resistance vessel radius even across conditions with different baseline levels of vascular conductance (Buckwalter and Clifford 2001; Thomas and Segal 2004). The magnitude of sympatholysis was calculated as the difference between the percentage change in FVC in response to sympathetic stimulation at rest and the percentage change in FVC in response to sympathetic stimulation during muscular contraction for control, Prazosin, and combined Prazosin + l‐NAME conditions (∆%FVC). All data are expressed as mean ± standard deviation.

### Statistics

The vasoconstrictor response to sympathetic stimulation was analyzed by three‐way repeated measures ANOVA (training group × muscle contractile state × drug condition; STATISTICA 10 Statsoft Inc., Tulsa, OK). The responses to each frequency of sympathetic stimulation were analyzed separately. The effects of exercise training and pharmacology on basal hemodynamics, muscle contractile force, exercise hyperemia, the response to selective α‐adrenoreceptor agonists, and the magnitude of sympatholysis were determined by two‐way repeated measures ANOVA (group × drug condition; SigmaPlot 12.3 Systat, Richmond, CA). Group differences in hindlimb skeletal muscle mass, body mass, heart mass, and the heart‐to‐body mass ratio were used as indices of training efficacy and were assessed by unpaired T‐test (SigmaPlot 12.3 Systat, Richmond, CA). When significant *F*‐ratios were detected, specific differences were assessed using Student–Newman–Keuls post hoc analysis. A *P*<0.05 was considered statistically significant.

## Results

### Training efficacy and basal hemodynamics

All exercise‐trained rats completed the 4 weeks of exercise training. Exercise‐trained rats had lower (*P *< 0.05) body mass and a higher (*P *< 0.05) heart mass : body mass ratio compared to sedentary rats (Table 1). Resting HR was lower (*P *< 0.05) in exercise‐trained compared to sedentary rats in all drug conditions, whereas resting MAP, FBF, and FVC were not different (*P *> 0.05) between exercise‐trained and sedentary rats (Table 2).

**Table 1:** Animal characteristics

Group	Body mass (g)	Heart mass (g)	Soleus mass (g)	Lateral gastrocnemius mass (g)	Medial gastrocnemius mass (g)	Heart‐to‐body mass ratio (mg g^‐1^)
Sedentary	451 ± 31	1.38 ± 0.13	0.21 ± 0.02	0.85 ± 0.08	1.54 ± 0.11	3.1 ± 0.2
Exercise trained	413 ± 30*	1.39 ± 0.08	0.21 ± 0.02	0.84 ± 0.13	1.52 ± 0.16	3.4 ± 0.2*

All values are mean ± SD. *indicates a statistically significant (*P *< 0.05) group difference.

**Table 2:** Basal hemodynamics

Group	Drug condition	HR (beats min^−1^)	MAP (mmHg)	FBF (mL min^−1^)	FVC (mL min^−1^ mmHg^−1^)
Sedentary	Control	404 ± 29	95 ± 8	4.0 ± 0.6	0.04 ± 0.006
	Prazosin	398 ± 23	84 ± 9^γ^	5.0 ± 0.7^γ^	0.061 ± 0.009^γ^
	Prazosin + l‐NAME	371 ± 18^γ^	124 ± 11^γ†^	5.5 ± 0.8^γ^	0.044 ± 0.006^†^
Exercise trained	Control	352 ± 31*	92 ± 11	3.8 ± 1.2	0.041 ± 0.011
	Prazosin	348 ± 24*	78 ± 11^γ^	4.6 ± 0.8^γ^	0.060 ± 0.012^γ^
	Prazosin + l‐NAME	342 ± 50*	116 ± 15^γ†^	5.0 ± 1.1^γ^	0.043 ± 0.008^†^

Baseline heart rate (HR), mean arterial pressure (MAP), femoral artery blood flow (FBF), and femoral vascular conductance (FVC) in Control, α_1_‐adrenergic blockade (Prazosin) and combined α_1_‐adrenergic and nitric oxide synthase blockade (l‐NAME) conditions. All values are mean ± SD. *indicates a significant difference from sedentary group within drug condition. ^γ^indicates a significant difference from control condition within group. ^†^indicates a significant difference between Prazosin and l‐NAME conditions within group. A *P *< 0.05 was considered statistically significant.

### Effect of exercise training on sympathetic vasoconstrictor responsiveness

**Figure 1:**
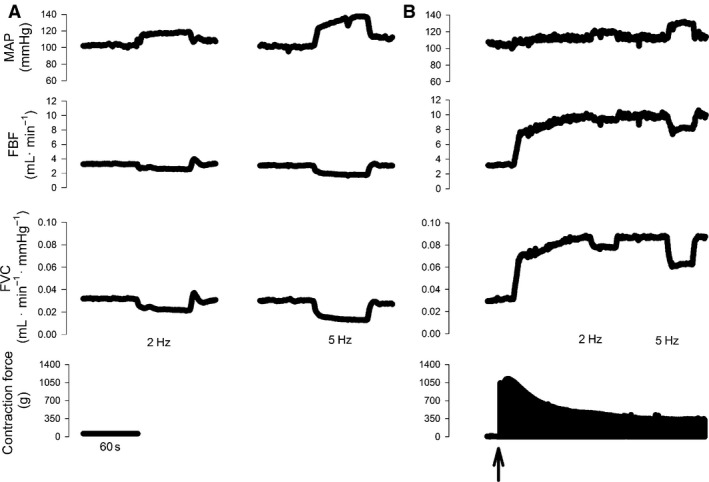
Original data from a representative sedentary rat illustrating the response of mean arterial blood pressure (MAP), femoral blood flow (FBF), femoral vascular conductance (FVC), and contractile force to lumbar sympathetic chain stimulation delivered at 2 and 5 Hz in resting skeletal muscle (A) and during skeletal muscle contraction at 60% of maximal contractile force (B).

**Figure 2:**
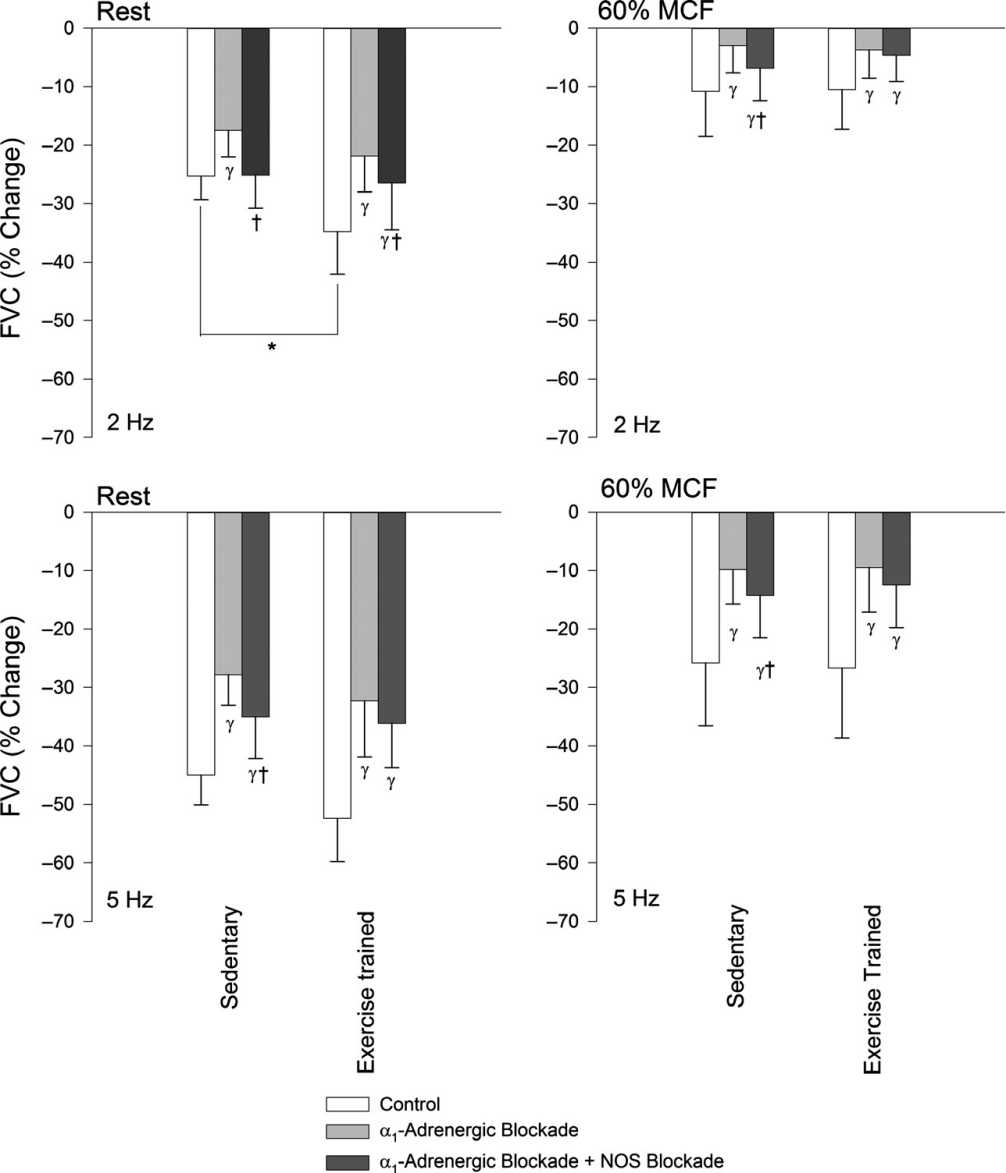
The percentage change of femoral vascular conductance (%FVC) in response to sympathetic stimulation delivered at 2 Hz (top) and 5 Hz (bottom) at rest (left) and during muscle contraction at 60% of maximal contractile force (MCF, right) in sedentary and exercise‐trained rats in Control (white), α_1_‐adrenoreceptor blockade with Prazosin (light gray; Prazosin, 20 *μ*g bolus, IV), and combined α_1_‐adrenoreceptor blockade and NOS blockade (dark gray; l‐NAME, 5 mg kg^−1^ IV) conditions. Values are mean ± SD. *indicates a significant difference between groups. γ indicates a significant difference from the control condition. †indicates a significant difference from Prazosin conditions within specified group. A *P *< 0.05 was considered statistically significant.

The response of HR, MAP, FBF, and FVC to sympathetic stimulation at rest and during muscle contraction in a representative rat is shown in Figure 1. At rest, sympathetic vasoconstrictor responsiveness was greater (*P *< 0.05) in exercise‐trained compared to sedentary rats in response to sympathetic stimulation delivered at 2 Hz, but was not different (*P *> 0.05) between groups in response to sympathetic stimulation delivered at 5 Hz (Fig. 2). Absolute changes in FBF and FVC in response to lumbar sympathetic chain stimulation are presented in Table 3.

**Table 3:** Absolute changes of femoral blood flow and vascular conductance in response to sympathetic stimulation at rest and during muscle contraction

Muscle contractile state	Group	Drug condition	2 Hz		5 Hz	
			FBF (mL min^−1^)	FVC (mL min^−1^ mmHg^−1^)	FBF (mL min^−1^)	FVC (mL min^−1^ mmHg^−1^)
Rest	Sedentary	Control	−0.9 ± 0.3	−0.011 ± 0.002	−1.5 ± 0.3	−0.019 ± 0.003
		Prazosin	−0.5 ± 0.3^γ^	−0.011 ± 0.003	−0.8 ± 0.4^γ^	−0.017 ± 0.002
		Prazosin + l‐NAME	−1.0 ± 0.3^†^	−0.011 ± 0.003	−1.5 ± 0.5^†^	−0.015 ± 0.003
	Exercise trained	Control	−1.1 ± 0.6	−0.014 ± 0.007	−1.6 ± 0.5	−0.021 ± 0.007
		Prazosin	−0.6 ± 0.3^γ^	−0.013 ± 0.004	−0.9 ± 0.3^γ^	−0.020 ± 0.008
		Prazosin + l‐NAME	−1.0 ± 0.4^†^	−0.011 ± 0.004^γ†^	−1.3 ± 0.5^†^	−0.015 ± 0.004^γ†^
Contraction	Sedentary	Control	−0.6 ± 0.6^‡^	−0.010 ± 0.007	−1.7 ± 0.9	−0.025 ± 0.008^‡^
		Prazosin	0.2 ± 0.5^γ‡^	−0.004 ± 0.005^γ‡^	−0.1 ± 0.7^γ‡^	−0.012 ± 0.006^γ^
		Prazosin + l‐NAME	−0.2 ± 0.6^γ†‡^	−0.007 ± 0.005^γ†‡^	−0.5 ± 0.5^γ‡^	−0.014 ± 0.006^γ^
	Exercise trained	Control	−0.3 ± 0.6^‡^	−0.011 ± 0.007^‡^	−1.4 ± 0.8	−0.028 ± 0.015^‡^
		Prazosin	0.2 ± 0.5^γ‡^	−0.004 ± 0.005^γ‡^	0.2 ± 0.7^γ‡^	−0.012 ± 0.009^γ‡^
		Prazosin + l‐NAME	0.2 ± 0.4^γ‡^	−0.005 ± 0.004^γ‡^	−0.1 ± 0.5^γ‡^	−0.014 ± 0.008^γ^

Absolute changes of femoral artery blood flow (FBF) and femoral vascular conductance (FVC) in response to sympathetic stimulation delivered at 2 and 5 Hz at rest and during muscle contraction at 60% maximal contractile force in Control, α_1_‐adrenergic blockade (Prazosin) and combined α_1_‐adrenergic and nitric oxide synthase blockade (l‐NAME) conditions. Values are mean ± SD. ^γ^indicates a difference from control conditions within the same group. ^†^indicates a significant difference between Prazosin and l‐NAME conditions within group. ^‡^indicates a significant difference between resting conditions and muscle contraction within the same group. A *P *< 0.05 was considered statistically significant.

**Figure 3:**
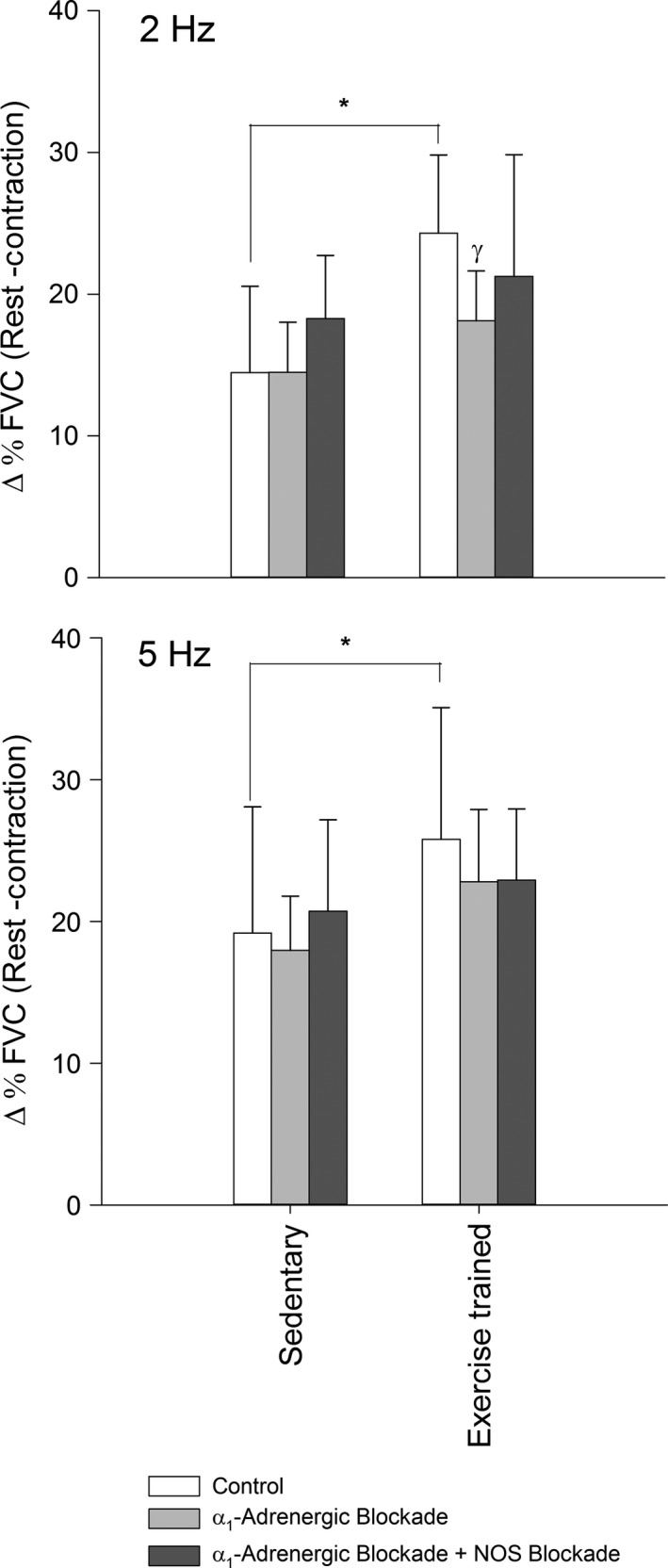
The magnitude of sympatholysis expressed as the difference between sympathetic vasoconstrictor responsiveness at rest and during muscular contraction (femoral vascular conductance, Δ%FVC) during 2 Hz (top) and 5 Hz (bottom) sympathetic stimulation in Control (open bars), α_1_‐adrenoreceptor blockade with Prazosin (20 *μ*g bolus, IV, gray bars), and combined α_1_‐adrenoreceptor blockade and NOS blockade (l‐NAME 5 mg kg^−1^ IV, dark gray bars) conditions in sedentary and exercise‐trained groups. Values are mean ± SD. *indicates a significant difference between groups. γindicates a significant difference from the control condition within group. A *P *< 0.05 was considered statistically significant.

During muscle contraction, sympathetic vasoconstrictor responsiveness was not different (*P *> 0.05) between exercise‐trained and sedentary rats (Fig. 2). The magnitude of sympatholysis was greater (*P *< 0.05) in exercise‐trained compared to sedentary rats (Fig. 3).

### Effects of α_1_‐adrenoreceptor blockade on sympathetic vasoconstrictor responsiveness

Blockade of α_1_‐adrenoreceptors reduced (*P *< 0.05) resting MAP and increased (*P *< 0.05) resting FBF and FVC by a similar (*P *> 0.05) magnitude in exercise‐trained and sedentary rats (Table 2).

Prazosin decreased (*P *< 0.05) resting vasoconstrictor responses to sympathetic stimulation delivered at both 2 Hz and 5 Hz in exercise‐trained and sedentary rats (Fig. 2) and the difference in resting vasoconstrictor responsiveness between exercise‐trained and sedentary rats was abolished by α_1_‐adrenoreceptor blockade.

In contracting muscle, Prazosin decreased (*P *< 0.05) evoked vasoconstrictor responses in both exercise‐trained and sedentary rats and sympathetic vasoconstrictor responsiveness was not different (*P *> 0.05) between groups in the presence of α_1_‐adrenoreceptors blockade.

Selective blockade of α_1_‐adrenoreceptors reduced sympatholysis in exercise‐trained rats when sympathetic stimulation was delivered at 2, but not 5 Hz (Fig. 3). Sympatholysis was not altered (*P *> 0.05) by blockade of α_1_‐adrenoreceptors in sedentary rats.

### Effects of combined α_1_‐adrenoreceptor and NOS blockade on sympathetic vasoconstrictor responsiveness

Combined blockade of α_1_‐adrenoreceptors and NOS increased (*P *< 0.05) MAP compared to the control and Prazosin conditions in exercise‐trained and sedentary rats. Resting HR was reduced (*P *< 0.05) in sedentary, but not (*P *> 0.05) exercise‐trained rats. However, HR remained lower (*P *< 0.05) in exercise‐trained compared to sedentary rats. FBF was not different (*P *> 0.05) from the Prazosin condition during combined α_1_‐adrenoreceptor and NOS inhibition in exercise‐trained and sedentary rats, whereas FVC was reduced (*P *< 0.05) to levels seen in the control condition in both groups (Table 2).

In resting skeletal muscle, combined blockade of α_1_‐adrenoreceptors and NOS increased (*P *< 0.05) vasoconstrictor responsiveness to sympathetic stimulation delivered at 2 and 5 Hz in sedentary rats (Fig. 2). In exercise‐trained rats, sympathetic vasoconstrictor responsiveness in resting muscle was increased in response to lumbar chain stimulation delivered at 2, but not 5 Hz following combined blockade of α_1_‐adrenoreceptors and NOS (Fig. 2).

During muscle contraction, combined α_1_‐adrenoreceptors and NOS blockade increased (*P *< 0.05) vasoconstrictor responsiveness to sympathetic stimulation delivered at both 2 and 5 Hz in sedentary rats. In contrast, sympathetic vasoconstrictor responsiveness was not altered (*P *> 0.05) by combined α_1_‐adrenoreceptors and NOS blockade during contraction in exercise‐trained rats.

The magnitude of sympatholysis was not different (*P *> 0.05) between the combined α_1_‐adrenoreceptor and NOS blockade and Prazosin conditions in exercise‐trained and sedentary rats (Fig. 3).

### Hyperemic response to contraction and muscle force production

The increase in FBF and FVC in response to muscle contraction was not different (*P *> 0.05) between exercise‐trained and sedentary rats during control conditions (Table 4). Following selective blockade of α_1_‐adrenoreceptors, the increase in FVC in response to contraction was augmented (*P *< 0.05) in both exercise‐trained and sedentary rats. The subsequent addition of NOS blockade reduced (*P *< 0.05) the increase in FVC in response to muscle contraction compared to the Prazosin condition in sedentary rats, whereas the increase in FVC in response to contraction was greater (*P *< 0.05) in exercise‐trained rats (Table 4).

**Table 4:** Hemodynamic response to muscle contraction

Group	Drug condition	HR (beats min^−1^)	MAP (mmHg)	FBF (mL min^−1^)	FVC (mL min^−1^ mmHg^−1^)
Sedentary	Control	−1 ± 10	5 ± 6	5.40 ± 1.39	0.051 ± 0.011
	Prazosin	20 ± 9^γ^	4 ± 4	5.70 ± 1.32	0.060 ± 0.010^γ^
	Prazosin + l‐NAME	10 ± 10^γ†^	−3 ± 4^γ†^	6.34 ± 1.33	0.056 ± 0.009^†^
Exercise trained	Control	5 ± 6	6 ± 4	5.96 ± 1.06	0.059 ± 0.013
	Prazosin	19 ± 21^γ^	4 ± 5	5.71 ± 1.36	0.065 ± 0.016^γ^
	Prazosin + l‐NAME	14 ± 14	−4 ± 5^γ†^	7.00 ± 2.14^γ †^	0.068 ± 0.020*^γ^

Absolute change of heart rate (HR), mean arterial pressure (MAP), femoral artery blood flow (FBF), and femoral artery vascular conductance (FVC) in response to muscle contraction at 60% of maximal contractile force in Control, α_1_‐adrenergic blockade (Prazosin) and combined α_1_‐adrenergic and nitric oxide synthase blockade (l‐NAME) conditions. Values are mean ± SD. *indicates a significant difference from sedentary group within drug condition. ^γ^indicates a difference from control conditions within a group. ^†^indicates a significant difference between Prazosin and l‐NAME conditions within a group. A *P *< 0.05 was considered statistically significant.

Muscle force production was greater (main effect; *P *< 0.05) in exercise‐trained (517 ± 127 g) compared to sedentary rats (417 ± 63 g). Muscle force production was not different (main effect; *P *> 0.05) between experimental conditions (Control: 454 ± 127 g; α_1_‐adrenoreceptor blockade: 458 ± 95 g α_1_‐adrenoreceptor and NOS blockade: 488 ± 97 g) and there was no interaction (*P *> 0.05) between experimental groups and conditions.

### Constrictor response to selective α‐adrenoreceptor agonists

Injection of phenylephrine and clonidine produced similar constrictor responses in exercise‐trained (Phenylephrine: −0.021 ± 0.010 mL·min^−1^·mmHg^−1^; Clonidine: −0.021 ± 0.009 mL·min^−1^·mmHg^−1^) and sedentary (Phenylephrine: −0.020 ± 0.005 mL·min^−1^·mmHg^−1^; Clonidine: −0.021 ± 0.005 mL·min^−1^·mmHg^−1^) rats in control conditions. Selective α_1_‐adrenoreceptor blockade abolished (*P *< 0.05) the constrictor response to phenylephrine in both exercise‐trained (0.000 ± 0.004 mL·min^−1^·mmHg^−1^) and sedentary rats (−0.002 ± 0.003 mL·min^−1^·mmHg^−1^), whereas the response to clonidine was not altered by Prazosin (exercise trained: −0.022 ± 0.008 mL·min^−1^·mmHg^−1^; sedentary: −0.026 ± 0.009 mL·min^−1^·mmHg^−1^).

## Discussion

The purpose of this study was to investigate the effect of exercise training on α_1_‐adrenoreceptor‐mediated vasoconstriction and NO‐mediated inhibition of sympathetic vasoconstriction at rest and during muscle contraction. Exercise training augmented α_1_‐adrenoreceptor‐mediated sympathetic vasoconstrictor responses to low‐, but not high‐, frequency sympathetic stimulation in resting skeletal muscle. Consistent with previous studies, exercise training enhanced sympatholysis (Jendzjowsky and DeLorey 2013a,b; Jendzjowsky et al. 2014; Mizuno et al. 2014). Selective blockade of α_1_‐adrenoreceptors abolished the improved sympatholysis in exercise‐trained rats, whereas NOS inhibition in combination with selective α_1_‐adrenoreceptor blockade did not alter sympatholysis. Our data demonstrate that the augmented sympatholysis following exercise training was mediated by greater inhibition of α_1_‐adrenoreceptor‐mediated vasoconstriction. Furthermore, functional α_1_‐adrenoreceptors were necessary for NO‐mediated inhibition of vasoconstriction, whereas NO was not required to inhibit α_2_‐adrenoreceptor‐ and nonadrenoreceptor‐mediated vasoconstriction in contracting skeletal muscle.

### Effect of α_1_‐adrenoreceptor blockade on sympathetic vasoconstrictor responsiveness in resting and contracting skeletal muscle

Consistent with previous studies from our laboratory (Jendzjowsky and DeLorey 2012, 2013a,b; Jendzjowsky et al. 2014), exercise training augmented sympathetic vasoconstrictor responsiveness to low‐frequency (2 Hz) sympathetic stimulation in the present study. In contrast to our previous studies, vasoconstrictor responsiveness to high‐frequency sympathetic stimulation was not increased following exercise training in the present study. Selective α_1_‐adrenoreceptor blockade reduced evoked vasoconstrictor responses to sympathetic stimulation at both low and high frequencies in sedentary and exercise‐trained rats. We have previously reported that α_2_‐adrenoreceptors do not contribute to evoked constrictor responses in sedentary male rats (Jendzjowsky and DeLorey 2013a). Therefore, in the present study the constrictor response to sympathetic stimulation in sedentary rats during selective α_1_‐adrenoreceptor blockade must be mediated by peptidergic and/or purinergic receptors. Consistent with this notion, tonic neuropeptide Y (NPY) – Y1 and purinergic receptor (P2X)‐mediated vasoconstriction, as well as evoked responses to selective agonists have been documented in resting skeletal muscle (Buckwalter et al. 2003, 2004a,b, 2005; Jackson et al. 2004, 2005; DeLorey et al. 2010, 2012). Recent evidence also suggests that NPY and P2X receptors regulate vasoconstriction on distal arterioles and activation of these receptors with selective agonists demonstrated that they are capable of producing substantial changes in vascular resistance (Al‐Khazraji et al. 2015).

In exercise‐trained rats, the augmented vasoconstrictor response to sympathetic stimulation was abolished by selective α_1_‐adrenoreceptor blockade suggesting that α_1_‐adrenoreceptors may become more responsive to low‐frequency sympathetic stimulation following exercise training. Consistent with augmented α_1_‐adrenoreceptor‐mediated vasoconstriction following exercise training, Svedenhag et al. (1991) reported that the pressor response to phenylephrine was augmented in endurance‐trained compared to untrained men. We have previously reported augmented α_2_‐adrenoreceptor‐mediated vasoconstriction following exercise training (Jendzjowsky and DeLorey 2013a). Thus, the combined data from this and our previous study (Jendzjowsky and DeLorey 2013a) indicate that exercise training augments both α_1_‐ and α_2_‐adrenoreceptor‐mediated vasoconstriction in resting skeletal muscle and adaptations in adrenoreceptor‐mediated vasoconstriction appear to be dependent on the frequency of sympathetic impulses/discharge.

During contraction, sympathetic vasoconstrictor responsiveness was blunted in both sedentary and exercise‐trained rats. The magnitude of contraction‐mediated inhibition of sympathetic vasoconstriction was greater in exercise‐trained compared to sedentary rats, consistent with previous finding from our laboratory (Jendzjowsky and DeLorey 2013a,b; Jendzjowsky et al. 2014). Other studies have also reported improved sympatholysis following exercise training (Mizuno et al. 2014; Mortensen et al. 2014). Selective blockade of α_1_‐adrenoreceptors did not alter sympatholysis in sedentary rats indicating that sympatholysis was not mediated by α_1_‐adrenoreceptors. Selective inhibition of α_1_‐adrenoreceptors blunted sympatholysis in exercise‐trained rats and the difference in the magnitude of sympatholysis between exercise‐trained and sedentary rats was abolished indicating that an improved blunting of α_1_‐adrenoreceptor‐mediated vasoconstriction contributes to the enhanced sympatholysis following exercise training. In a recent study (Jendzjowsky and DeLorey 2013a), we reported that selective α_2_‐adrenoreceptor blockade decreased sympatholysis in heavy‐intensity exercise‐trained rats. However, the magnitude of sympatholysis remained significantly greater in heavy‐intensity‐trained compared to sedentary rats demonstrating that improved blunting of α_2_‐adrenoreceptor constrictor responses only mediated a portion of the training‐mediated enhancement of sympatholysis (Jendzjowsky and DeLorey 2013a). The findings from the present and our recent study (Jendzjowsky and DeLorey 2013a) indicate that heavy‐intensity exercise training improves blunting of both α_1_‐ and α_2_‐adrenoreceptor‐mediated vasoconstriction during exercise.

### Effect of combined α_1_‐adrenoreceptor blockade and NOS inhibition on sympathetic vasoconstrictor responsiveness in resting and contracting skeletal muscle

In sedentary rats at rest, the addition of NOS blockade to selective α_1_‐adrenoreceptor blockade (combined blockade) increased evoked constrictor responses to sympathetic stimulation delivered at both 2 and 5 Hz. The present data and our earlier finding that α_2_‐adrenoreceptors do not contribute to evoked vasoconstriction at rest (Jendzjowsky and DeLorey 2013a) suggest that NO blunts nonadrenoreceptor‐mediated vasoconstriction in resting of skeletal muscle of sedentary rats. In exercise‐trained rats, combined inhibition of NOS and α_1_‐adrenoreceptors increased the constrictor response to sympathetic stimulation delivered at 2 Hz, whereas the constrictor response to stimulation delivered at 5 Hz was not statistically different from the selective α_1_ blockade condition. Thus, in exercise‐trained rats NO inhibits α_2_‐adrenoreceptors and nonadrenoreceptor‐mediated constrictor responses to low‐frequency stimulation of the sympathetic chain.

During contraction, combined blockade of NOS and α_1_‐adrenoreceptors increased sympathetic vasoconstrictor responsiveness in sedentary rats. The magnitude of sympatholysis was not altered by combined NOS inhibition and selective α_1_‐adrenoreceptor blockade suggesting that NO is not required for contraction‐mediated inhibition of sympathetic vasoconstriction in sedentary rats. In exercise‐trained rats, combined NOS inhibition and α_1_‐adrenoreceptor blockade did not alter sympathetic vasoconstrictor responsiveness during contraction, suggesting that in exercise‐trained rats α_1_‐adrenoreceptors are required for NO‐mediated inhibition of sympathetic vasoconstriction during contraction. We have previously shown that in the presence of selective α_2_‐adrenoreceptor blockade, NOS inhibition significantly reduced the magnitude of sympatholysis in heavy‐intensity exercise‐trained rats suggesting that exercise training augmented NO‐mediated inhibition of α_1_‐adrenoreceptors (Jendzjowsky and DeLorey 2013a). Buckwalter et al. (2004c) have also reported that NOS blockade abolished contraction‐mediated inhibition of α_1_‐adrenoreceptors during heavy‐intensity exercise. The lack of difference in the magnitude of sympatholysis between the selective α_1_‐adrenoreceptor blockade condition and the combined α_1_‐adrenoreceptor and NOS blockade condition in exercise‐trained rats in the present study further indicates that α_1_‐adrenoreceptors are required for NO‐mediated sympatholysis in exercise‐trained male rats. Collectively, the data from our present and recent studies indicate that exercise training enhances sympatholysis through an NO‐dependent inhibition of α_1_‐adrenoreceptor‐mediated vasoconstriction. α_1_‐adrenoreceptors are G‐protein‐coupled receptors that produce vasoconstriction through a signaling cascade where binding of NE leads to increased phospholipase c, diacylglycerol, and inositol triphosphate activity that evokes the release of Ca^++^ from the sarcoplasmic reticulum and influx of extracellular Ca^++^ through TRPC3 and TPRC6 channels (Minneman 1988; Gohla et al. 2000; Westcott and Segal 2013). Further investigation will be required to determine how NO interacts with this signaling pathway to alter intracellular [Ca^++^] and vascular smooth muscle contraction.

The data from the present study also suggest that NO was not required to inhibit α_2_‐adrenoreceptor‐ and nonadrenoreceptor‐mediated vasoconstriction during exercise in sedentary and exercise‐trained rats. In agreement with our data, NOS inhibition did not impair the blunting of α_2_‐adrenoreceptor‐mediated vasoconstriction during moderate‐ or heavy‐intensity exercise in dogs (Buckwalter et al. 2004c). In humans, vasoconstrictor responses to selective α_1_‐ and α_2_‐adrenoreceptor agonists during moderate‐intensity handgrip exercise were also not altered by inhibition of NOS production (Dinenno and Joyner 2003).

Purinergic and NPY‐Y1 receptor‐mediated vasoconstriction is also inhibited during exercise (Buckwalter et al. 2004b, 2005; DeLorey et al. 2010, 2012). Acidosis and elevated temperature have been shown to reduce the responsiveness of purinergic receptors in vitro (Kluess et al. 2005a,b), however NOS blockade did not alter NPY‐Y1 receptor responsiveness during exercise in canines (Buckwalter et al. 2005). To our knowledge, the effect of exercise training on peptidergic and purinergic receptor‐mediated vasoconstriction has not been investigated and further investigation in this area appears warranted.

### Perspectives

In a series of recent studies, our laboratory has investigated the effects of exercise training on sympathetic vascular control in resting and contracting skeletal muscle (Jendzjowsky and DeLorey 2012, 2013a,b; Jendzjowsky et al. 2014). Our first studies demonstrated that exercise training augmented sympathetic vasoconstrictor responsiveness (Jendzjowsky and DeLorey 2012) and enhanced sympatholysis through an NO‐dependent mechanism (Jendzjowsky and DeLorey 2013b). Subsequently, we reported that exercise training altered the relative contributions of postsynaptic α‐adrenoreceptors to evoked vasoconstrictor responses and that α_2_‐adrenoreceptor‐mediated vasoconstriction was augmented in resting and contracting skeletal muscle following exercise training (Jendzjowsky and DeLorey 2013a). Our data also suggested that the blunting of α_1_‐adrenoreceptor vasoconstriction may underlie the enhanced sympatholysis following exercise training. In the present study, selective α_1_‐adrenoreceptor blockade abolished exercise training‐induced improvements in sympatholysis, further indicating that the enhanced sympatholysis following exercise training is dependent on inhibition of α_1_‐adrenoreceptor‐mediated vasoconstriction.

Earlier studies have suggested that α_2_‐adrenoreceptors were primarily expressed on distal arterioles positioning them in close proximity to active muscle fibers where they may be readily inhibited by local sympatholytic molecules, allowing α_2_‐adrenoreceptors to precisely regulate the distribution of blood flow between and within muscles. In contrast, α_1_‐adrenoreceptors were positioned upstream on larger arterioles and remained relatively resistant to inhibition ensuring that vasoconstriction remained present in active muscles to maintain systemic blood pressure (Faber 1988; Ohyanagi et al. 1991; Buckwalter and Clifford 2001; Thomas and Segal 2004). Recent studies have challenged this model of receptor distribution and suggest that the potential role of individual postsynaptic receptors in sympatholysis may need to be reconsidered. In the mouse gluteus maximus muscle, α_2_‐adrenoreceptors appear to predominately regulate vasoconstriction in proximal 1A arterioles, whereas α_1_‐adrenoreceptors appear to regulate vasoconstriction in more distal 3A arterioles (Moore et al. 2010). In agreement, a recent study by Al‐Khazraji et al. (2015) in the rat gluteus maximus muscle suggests that α‐adrenoreceptors primarily regulate resistance in proximal arterioles (branch order 1A to 3A), whereas purinergic and peptidergic receptors constrict more distal arteries (branch orders 4A and 5A) (Al‐Khazraji et al. 2015). If we accept the premise that receptors on distal arteries are more susceptible to metabolic inhibition, then the present demonstration of blunted α_1_‐adrenoreceptor responsiveness following exercise training suggests that α_1_‐adrenoreceptors are expressed on distal arteries in the hindlimb vasculature of male rats.

It is also possible that exercise training induces changes in the expression and/or distribution of α‐adrenoreceptors. Determination of the expression and distribution of postsynaptic sympathetic receptors is beyond the scope of the present investigation. However, advancing our understanding of the effects of exercise training on receptor expression and distribution will be essential to establish a complete understanding of the effects of chronic endurance exercise training on sympathetic vascular control in health and disease.

## Conclusion

In conclusion, this study demonstrated that the augmented sympatholysis following exercise training was mediated by greater inhibition of α_1_‐adrenoreceptor‐mediated vasoconstriction. Furthermore, functional α_1_‐adrenoreceptors were necessary for NO‐mediated inhibition of vasoconstriction, whereas NO was not required to inhibit α_2_‐adrenoreceptor‐ and nonadrenoreceptor‐mediated vasoconstriction in contracting skeletal muscle.

## Conflict of Interest

None declared.
